# Ovulation suppression following subcutaneous administration of depot medroxyprogesterone acetate

**DOI:** 10.1016/j.conx.2022.100073

**Published:** 2022-02-23

**Authors:** Douglas J. Taylor, Vera Halpern, Vivian Brache, Luis Bahamondes, Jeffrey T. Jensen, Laneta J. Dorflinger

**Affiliations:** a FHI 360, Durham, NC, United States; b Profamilia, Biomedical Research Department, Santo Domingo, Dominican Republic; c Department of Obstetrics and Gynecology, University of Campinas Faculty of Medical Sciences, Campinas, Brazil; d Department of Obstetrics and Gynecology, Oregon Health and Science University, Portland, OR, United States

**Keywords:** DMPA, Depo-Provera, Depo-subQ Provera, Pharmacokinetics, Pharmacodynamics

## Abstract

**Objectives:**

To characterize the relationship between serum medroxyprogesterone acetate (MPA) concentrations and ovulation suppression, and to estimate the risk of ovulation for investigational subcutaneous regimens of Depo-Provera CI (Depo-Provera) and Depo-subQ Provera 104 (Depo-subQ).

**Study Design:**

We performed a secondary analysis of 2 studies that assessed the pharmacokinetics and pharmacodynamics of MPA when Depo-Provera is administered subcutaneously rather than by the labeled intramuscular route. Each woman received a single 45 mg to 300 mg subcutaneous injection of Depo-Provera, a single 104 mg subcutaneous injection of Depo-subQ, or 2 injections of Depo-subQ at 3-month intervals. We used an elevation of serum progesterone ≥4.7 ng/mL as a surrogate for ovulation and non-parametric statistical methods to assess pharmacokinetic and pharmacodynamic relationships.

**Results:**

This analysis included 101 women with body mass index (BMI) 18 to 34 kg/m^2^. Return of ovulation occurred at a median MPA concentration of 0.07 ng/mL (95% CI: 0.06–0.08) and the 90th percentile was 0.10 ng/mL (95% CI: 0.09–0.14). Neither age, race, nor BMI significantly influenced this relationship. The estimated probabilities of ovulation within 4 months of a 104 mg subcutaneous injection and within 7 months of a 150 mg subcutaneous injection (6 plus a 1-month grace) were each below 2.2%.

**Conclusions:**

The typical MPA concentration associated with loss of ovulation suppression is substantially less than the commonly cited threshold of 0.2 ng/mL. Based on our results, MPA levels would rarely be low enough to permit ovulation if the Depo-subQ reinjection interval were extended to four months or if 150 mg Depo-Provera were injected subcutaneously every 6 months.

**Implications:**

Extending the three-month Depo-subQ reinjection interval by one month would result in a 25% reduction in yearly MPA exposure, with little risk of pregnancy. Off-label subcutaneous administration of 150 mg Depo-Provera every 6 months would be a highly effective repurposing of an excellent product, with a similar reduction in cumulative exposure.

## 1 Introduction

The intramuscular injectable Depo-Provera CI (150 mg depot medroxyprogesterone acetate [MPA] in 1 mL suspension) (Depo-Provera) is a highly effective contraceptive method, with a pregnancy rate of 0.3 per 100 person-years when injected every 3 months [1]. A lower dose subcutaneous formulation supplied in a pre-filled glass syringe, Depo-subQ Provera 104 (104 mg MPA in 0.65 mL suspension) (Depo-subQ), is also injected every 3 months. Despite a 31% lower dose, Depo-subQ achieves comparable efficacy to Depo-Provera due to slower drug absorption for the subcutaneous route of administration [1]. Depo-subQ is also associated with a similar side effects profile as Depo-Provera, including bleeding disturbances, weight gain, reduced mineral bone density, and delayed return to fertility [2], [3], [4].

Prescribing information for Depo-subQ emphasizes the need to re-inject every 3 months, noting the risk of ovulation as early as 14 weeks after initiating treatment [4]. Based on the pharmacokinetics (PK) and pharmacodynamics (PD) studies that supported approval of Depo-subQ in the USA and Sayana Press (the same drug pre-filled in a Uniject delivery system) in Europe, a serum progesterone concentration ≥4.7 ng/mL (the primary surrogate for ovulation used in the studies) may occur still earlier [5], [6], [7], [8]. These events were typically attributed to an unexpected pharmacokinetics profile (as might occur with an incomplete injection) or inconsistent with actual ovulation based on supportive gonadotropic hormone data. Regardless, the risk of an early ovulation is clearly low, and accumulation of MPA levels over 1 to 2 years of use implies that the residual chance of ovulation decreases (and the delay in return to fertility increases) with continued use of the method [3].

The side effect profile coupled with a lack of appreciable ovulation risk for Depo-subQ has led various researchers to consider regimens that could further reduce MPA exposure [9]. Here we present a secondary analysis of 2 contemporary pharmacokinetics and pharmacodynamics studies designed to inform such strategies [10,11]. Our initial aim was to characterize the distribution of serum MPA concentrations when ovulation returns. We leveraged this relationship to gain further insight into the contraceptive potential of 2 specific approaches to reducing MPA exposure by approximately 25% compared to the current subcutaneous regimen: extending the Depo-subQ injection interval from 3 to 4 months and repurposing the existing 3-month Depo-Provera intramuscular product as a 6-month subcutaneous method.

## 2 Materials and methods

We performed a secondary analysis of 2 contemporary pharmacokinetics and pharmacodynamics studies of subcutaneously administered MPA conducted between September 2015 and May 2018 [10,11]. Briefly, FHI 360 Study number 702179 (Clinical Trial Registration Number NCT02456584) randomized 36 women from sites in the Dominican Republic and the USA to receive a single 150 mg (1 mL) or 300 mg (2 mL) subcutaneous injection of Depo-Provera, or two 104 mg (0.65 mL) subcutaneous injections of Depo-subQ at 3-month intervals, in a 2:1:1 ratio. An additional 6 women were purposefully assigned to the 150 mg group, and one withdrew consent, leaving 41 treated subjects. Women were followed for a minimum of 7.5 months and then until ovulation was detected, but for a maximum of 18 months. We measured serum progesterone concentrations weekly starting 12 weeks after each injection. Participants who did not have an elevated progesterone concentration ≥4.7 ng/mL within 12 months of treatment initiation returned for weekly progesterone assessments in month 15 and (if still anovulatory) again in month 18 (the visit schedule within 12 weeks of injection and supportive pharmacodynamic measures are detailed elsewhere [10]). FHI 360 Study number 834119 (NCT02732418) randomized 60 participants in the Dominican Republic, Chile, and Brazil to receive a single 45 mg (0.3 mL), 75 mg (0.5 mL), or 105 mg (0.7 mL) subcutaneous injection of Depo-Provera, or a single 104 mg subcutaneous injection of Depo-subQ, in a 1:1:1:1 ratio [11]. We measured serum progesterone levels at least weekly until ovulation was confirmed or month 7.5, whichever was earlier. However, all Study 834119 participants contributed a full 7.5 months of MPA testing, regardless of ovulation status (details of the testing algorithms, including other pharmacodynamic measures, are described elsewhere [11]).

In both studies, Depo-Provera was drawn from 1 mL vials and delivered subcutaneously through the same gauge and length needle as the Depo-subQ pre-filled syringe (26 G × 3/8″); treatment was initiated within the first five days of menses; and all injections were given subcutaneously in the abdomen. Serum MPA concentrations were determined using a high-performance liquid chromatography-mass spectrometry assay at a central laboratory (PPD Development; Richmond, VA) with accuracy −3.0% to 3.7% and interassay CV of 4.5% to 10.5%. Progesterone concentrations were determined at local laboratories using commercial electrochemiluminescence immunoassays (Roche Elecsys 2010, Roche Cobas e411, and Siemens Advia Centaur) with sensitivity ≤0.05 ng/mL and interassay CV less than 10%. The FHI 360 Protection of Human Subjects Committee and ethics boards applicable to each research site approved the studies.

One noteworthy difference between the two protocols was that Study 702179 used a single elevated progesterone ≥4.7 ng/mL as a surrogate for ovulation while Study 834119 incorporated follicular rupture in the ovulation algorithm. Here we define presumptive ovulation as a single elevated progesterone ≥4.7 ng/mL to harmonize measures between studies and with historical data. A second important difference was that Study 702179 exited participants when ovulation was detected. Accounting for this type of informative censoring when jointly modeling longitudinal MPA and time to ovulation data with nonlinear mixed-effects (population PK/PD) models requires making additional assumptions for already complex analysis methods [12]. We instead used a nonparametric, 2-stage estimation procedure to minimize assumptions regarding drug absorption rates, censoring mechanisms, and distributions of random variables.

In stage 1, we estimated each participant's underlying pharmacokinetics profile based on their rich sample of MPA measurements (a minimum of 23 specimens over the first 7.5 months of follow-up) using locally re-weighted nonparametric regression (LOESS), which accounts for measurement error and potential outliers in observed values over time [13]. We used these profiles to predict each participant's MPA concentration on the last day when elevated progesterone could be ruled out and the first day that an elevated progesterone was detected. This interval, which ranged from 2 to 9 days in length among participants who ovulated before month 12, was fixed at 56 days for participants in Study 702179 who did not have ovulation detected until month 15 or 18, since up to two 28-day menstrual cycles could have been missed in the testing gaps after month 12. Participants who never had ovulation detected had their MPA levels censored at the maximum of the concentration predicted for the day they ended testing and the lower limit of quantification (0.02 ng/mL). In stage 2, we estimated the cumulative distribution of MPA concentrations when ovulation returned by applying Turnbull's nonparametric maximum likelihood algorithm to the censored stage 1 data [14]. Distributions of MPA concentrations at other relevant timepoints (e.g., 7 months after a 150 mg dose) were similarly obtained. We used parametric alternatives to Turnbull's algorithm, selected based on Bayesian Information Criteria, to compute bootstrap confidence intervals (CI), estimate covariate effects, and perform sensitivity analyses.

If we can assume that the MPA concentration required to suppress an individual's ovulation (denoted by the random variable *Y_ov_*) does not vary with time or extent of exposure, then the probability they ovulate within *T* months of injection is simply the probability that their MPA level falls below *Y_ov_* on or before month *T*. The probability of this occurring can be approximated by the following equation:

(Equation 1) $$pr\left\{X_{T}<Y_{OV}\right\}=\int_{0}^{\infty }F_{X}\left(y\right)dF_{Y}\left(y\right),$$

where *F_X_*(*•*) is the cumulative distribution of the MPA concentration at month *T* (denoted *X_T_*) and *F_Y_*(*•*) is the cumulative distribution of *Y_ov_*. Since MPA concentrations may not uniformly decrease with time since injection, it would be more accurate to replace the distribution of MPA at month *T* in Eq. (1) with the distribution of the minimum concentration on or before month *T* (*X_min_*). However, this distinction was not of practical importance for the formulations, doses, and time periods relevant to our analyses. Using *X_T_* in place of *X_min_* also facilitates finding a solution when – contrary to our setting – MPA data are sparse and the distribution of *X_min_* cannot be reliably obtained. We solved Eq. (1) using both nonparametric and parametric methods when estimating the probability of ovulation within four months of a 104 mg injection and within seven months (6 plus a 1-month grace) of a 150 mg injection. Additional details are in the supplemental Appendix. All analyses were performed using SAS/STAT or SAS/IML Version 9.4.

## 3 Results

### 3.1 Demographics and participant disposition

Detailed characteristics of the 101 participants contributing to our analyses are in the previous manuscripts [10,11]. Briefly, the median age was 34 years (range 22–40); 35.6% self-identified as white and 64.4% identified as black or biracial; and the median body mass index (BMI; kg/m^2^) was 26.6 (range 18.1–33.9; Table 1). Fifty-nine participants (58.4%) had ovulation detected during follow-up. We excluded 6 participants (5.9%) from pharmacokinetics analyses due to elevated MPA in their baseline specimens and one (1.0%) from pharmacodynamics analyses due to the use of a medication which may have impacted ovulatory function.

**Table 1:** Demographic characteristics and participant disposition in two trials conducted between 2015 and 2018 that assessed the pharmacokinetics and pharmacodynamics of MPA when Depo-Provera is administered subcutaneously rather than by the labeled intramuscular route [10,11]^1^

	Study 702179(n = 41)	Study 834119(n = 60)	Total(n = 101)
Age (years)			
Median (Range)	34 (25–39)	33 (22–40)	34 (22–40)
> 35	16 (39.0%)	24 (40.0%)	40 (39.6%)
Race			
White	3 (7.3%)	33 (55.0%)	36 (35.6%)
Biracial^2^ or black	38 (92.7%)	27 (45.0%)	65 (64.4%)
BMI (kg/m^2^)			
Median (Range)	26.6 (18.1–32.8)	26.7 (19.0–33.9)	26.6 (18.1–33.9)
< 25	13 (31.7%)	21 (35.0%)	34 (33.7%)
25 to 30	18 (43.9%)	25 (41.7%)	43 (42.6%)
> 30	10 (24.4%)	14 (23.3%)	24 (23.8%)
Study disposition			
Completed follow-up	41 (100%)	54 (90.0%)	95 (94.1%)
Ovulated	35 (85.4%)^3^	24 (40.0%)	59 (58.4%)

1 Study 702179 [10] was conducted in the Dominican Republic and the USA. Study 834119 [11] was conducted in Brazil, Chile, and the Dominican Republic. Data presented are n (%) unless otherwise noted.

2 Greater than 90% of participants identifying as biracial were of African and European descent.

3 Includes 16 ovulations detected after the primary 12-month follow-up period.

### 3.2 Pharmacokinetics and pharmacodynamics data

**Fig. 1:**
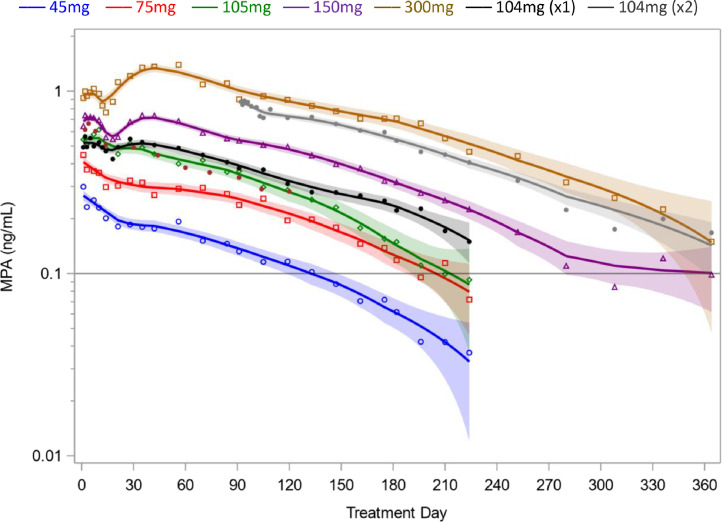
Geometric mean MPA concentrations following subcutaneous administration of 45–300 mg Depo-Provera or 104 mg Depo-subQ in two trials conducted between 2015 and 2018 [10,11]. Solid lines and 95% confidence bands are based on locally re-weighted nonparametric regression. The first three months of data were pooled for participants receiving one (x1) or two (x2) injections of Depo-subQ. Abbreviation: MPA, medroxyprogesterone acetate.

The 150 mg and 300 mg dose groups exhibited pronounced bi-phasic drug absorption on average, with a secondary peak after day 30 (Fig. 1). Individual pharmacokinetic profiles were decidedly heterogeneous, variously exhibiting burst effects, near zero-order absorption for extended time periods, and other complex absorption patterns (Supplemental Fig. S1). The geometric mean (GM) maximum concentration (C_max_) ranged from 0.35 ng/mL (45 mg) to 1.74 ng/mL (300 mg), with the increase proportional to dose in the 45mg to 150mg range (supplemental Table S1). Geometric mean MPA levels were generally higher for 104 mg Depo-subQ than for subcutaneous administration of 105 mg Depo-Provera, due in part to one participant in the 105 mg group with unusually low levels throughout follow-up (PN 2321, Supplemental Fig. S1). Geometric mean concentrations were proportional to dose between month 1 and month 4, but decreased more rapidly thereafter in the 45 mg, 75 mg, and 105 mg groups (the same is apparent when considering the GM of the minimum MPA concentration on or before month *T*; supplemental Table S2). Average MPA levels appeared to plateau near 0.1 ng/mL after month 9 in the 150 mg group, consistent with informative censoring due to discontinuing participants when ovulation occurred in Study 702179.

**Fig. 2:**
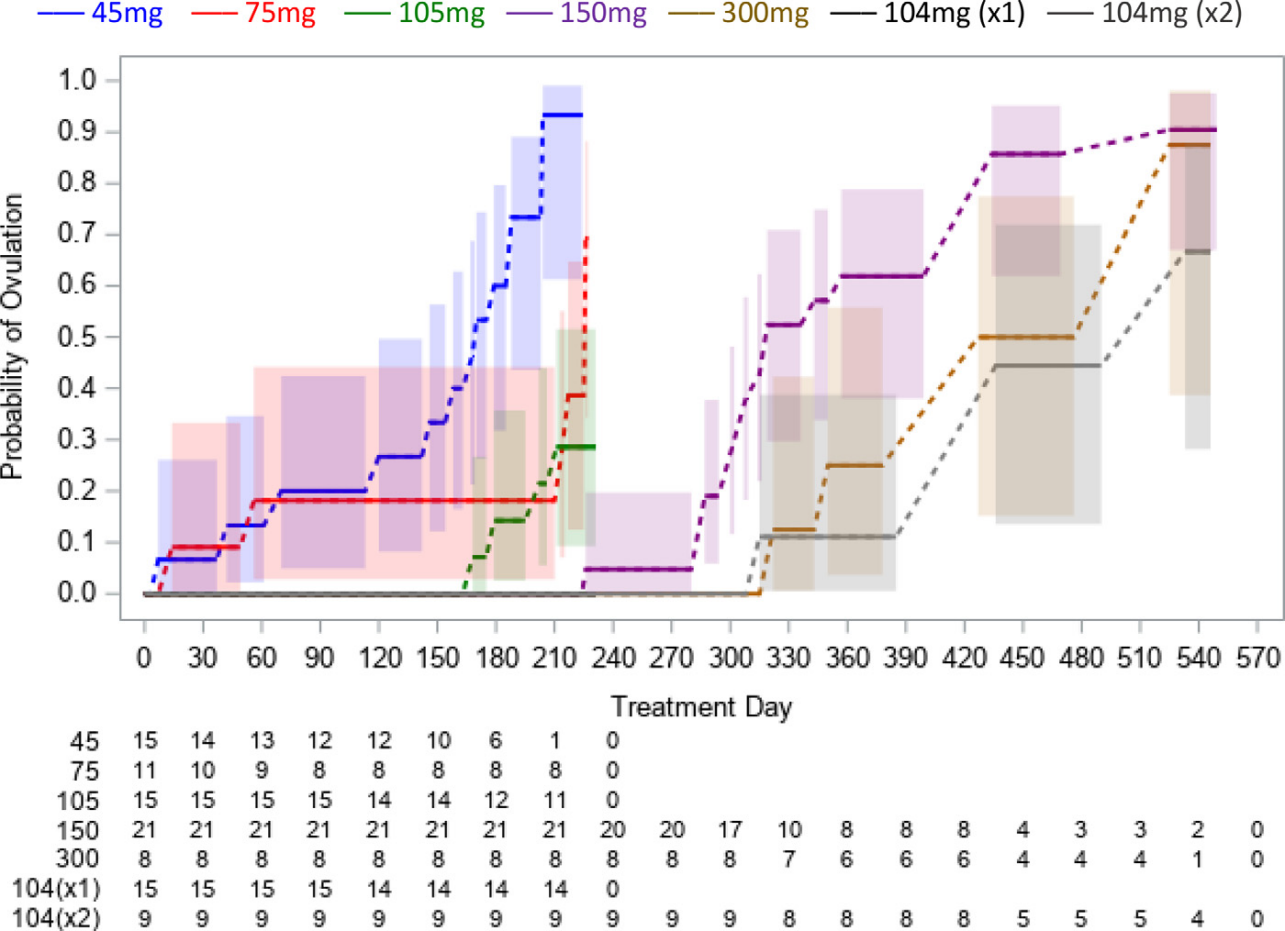
Cumulative probability of ovulation (progesterone ≥4.7 ng/mL) following subcutaneous administration of 45–300 mg Depo-Provera or 104 mg Depo-subQ in two trials conducted between 2015 and 2018 [10,11]. Shaded regions are 95% confidence bands. Numbers at-risk are below the x-axis. There were no events among subjects who received a single (x1) dose of Depo-subQ.

None of the 15 participants who received a single subcutaneous injection of 104 mg Depo-subQ ovulated during 7.5 months of follow-up, and only 1 of 9 who received a second dose at month three ovulated within 9 months of their second injection. The earliest ovulation among participants who received a 45 mg, 75 mg, 105 mg, 150 mg, or 300 mg subcutaneous injection of Depo-Provera occurred at month 0.2, 0.5, 5.5, 7.5, and 11, respectively (Fig. 2).

### 3.3 Serum MPA concentrations and ovulation suppression

**Fig. 3:**
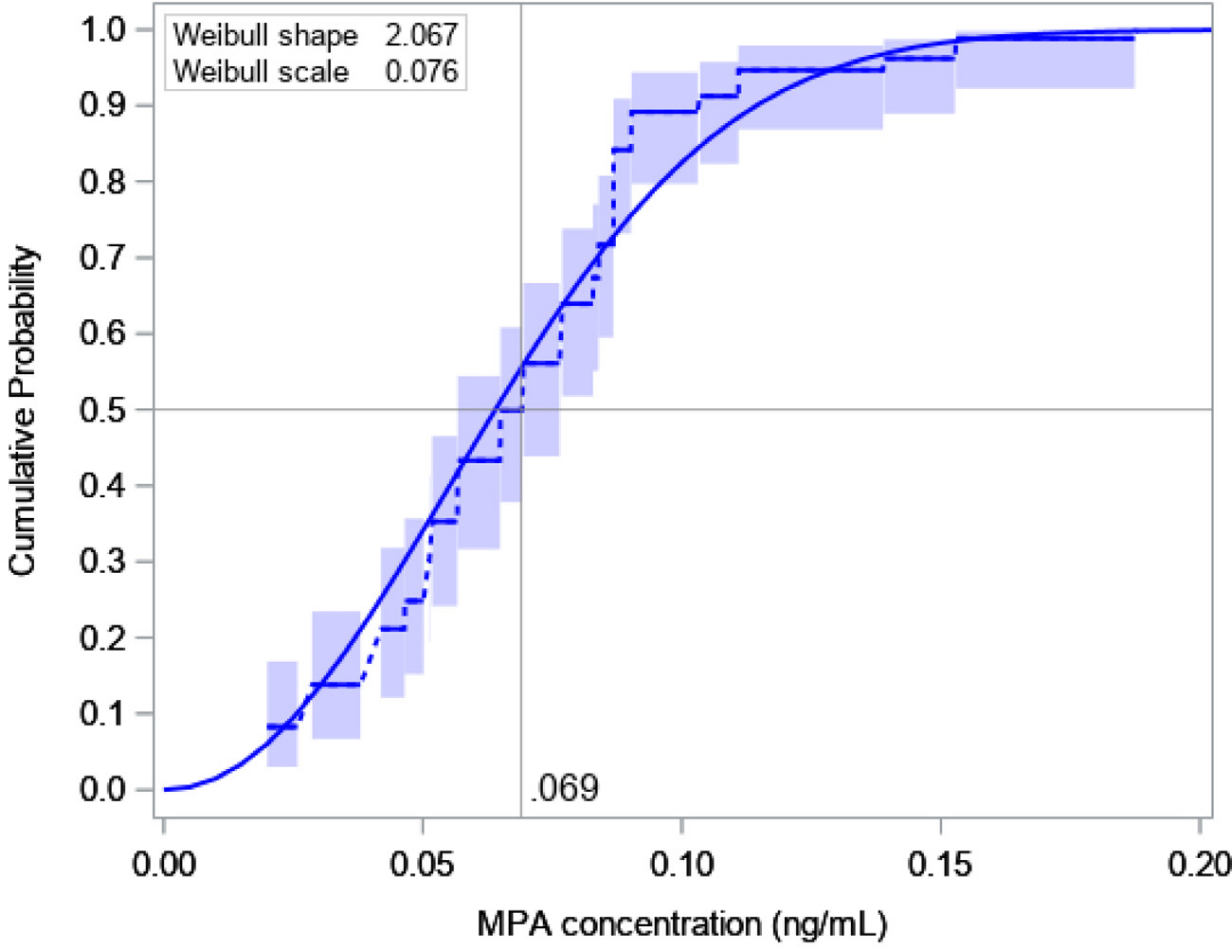
Cumulative distribution of the MPA concentration when ovulation returns (progesterone ≥ 4.7 ng/mL) following subcutaneous administration of 45–300 mg Depo-Provera or 104 mg Depo-subQ in two trials conducted between 2015 and 2018 [10,11]. Dashed lines and 95% confidence bands are based on non-parametric maximum likelihood estimation. Solid curve is Weibull model fit, which was used to estimate covariate effects in Table 2. Abbreviation: MPA, medroxyprogesterone acetate.

The LOESS-predicted MPA concentrations at timepoints relevant to analyses exhibited excellent fits to the data (Supplemental Fig. S2). The nonparametric estimate of the median MPA concentration when ovulation returned was 0.07 ng/mL (95% CI: 0.06–0.08), the 90th percentile was 0.10 ng/mL (95% CI: 0.09–0.14), and no participants ovulated at concentrations exceeding 0.2 ng/mL (Fig. 3). Assuming *Y_ov_* was sampled from a Weibull distribution led to similar results (median: 0.06 ng/mL; 90th percentile: 0.11 ng/mL). Neither study, age, race, BMI, nor administered dose were significantly associated with the MPA concentration when ovulation returned, but a faster rate of MPA decline in the 2 to 4 weeks prior to ovulation was associated with lower MPA levels when progesterone became elevated (*p* = 0.02; Table 2).

**Table 2:** Median MPA concentration (ng/mL) when ovulation returns following subcutaneous administration of 45–300 mg Depo-Provera or 104 mg Depo-subQ in two trials conducted between 2015 and 2018 [10,11]. All results are based on a Weibull distribution assumption.

	N	Median (95% CI)	*p*value
Overall	98	0.064 (0.055, 0.074)	NA
Study number			
702179	41	0.065 (0.055, 0.077)	
834119	57	0.062 (0.051, 0.076)	
BMI (kg/m^2^)			0.36
<25	33	0.059 (0.048, 0.072)	
25–30	42	0.070 (0.058, 0.084)	
>30	23	0.062 (0.048, 0.079)	
Age (years)			0.87
≤35	59	0.064 (0.054, 0.075)	
>35	39	0.065 (0.053, 0.080)	
Race			0.09
Black/biracial	64	0.068 (0.058, 0.079)	
White	34	0.054 (0.043, 0.069)	
Half-life (days)^a^			0.02
<45	33	0.055 (0.045, 0.067)	
45–90	31	0.064 (0.053, 0.078)	
>90	34	0.079 (0.064, 0.097)	
Dose of MPA	98	NA	0.37

MPA, medroxyprogesterone acetate.

a Corresponding to rate of MPA decrease in the 2–4 weeks prior to last non-elevated progesterone measurement.

### 3.4 Probability of ovulation within 4 or 7 months of a 104 mg or 150 mg injection

**Fig. 4:**
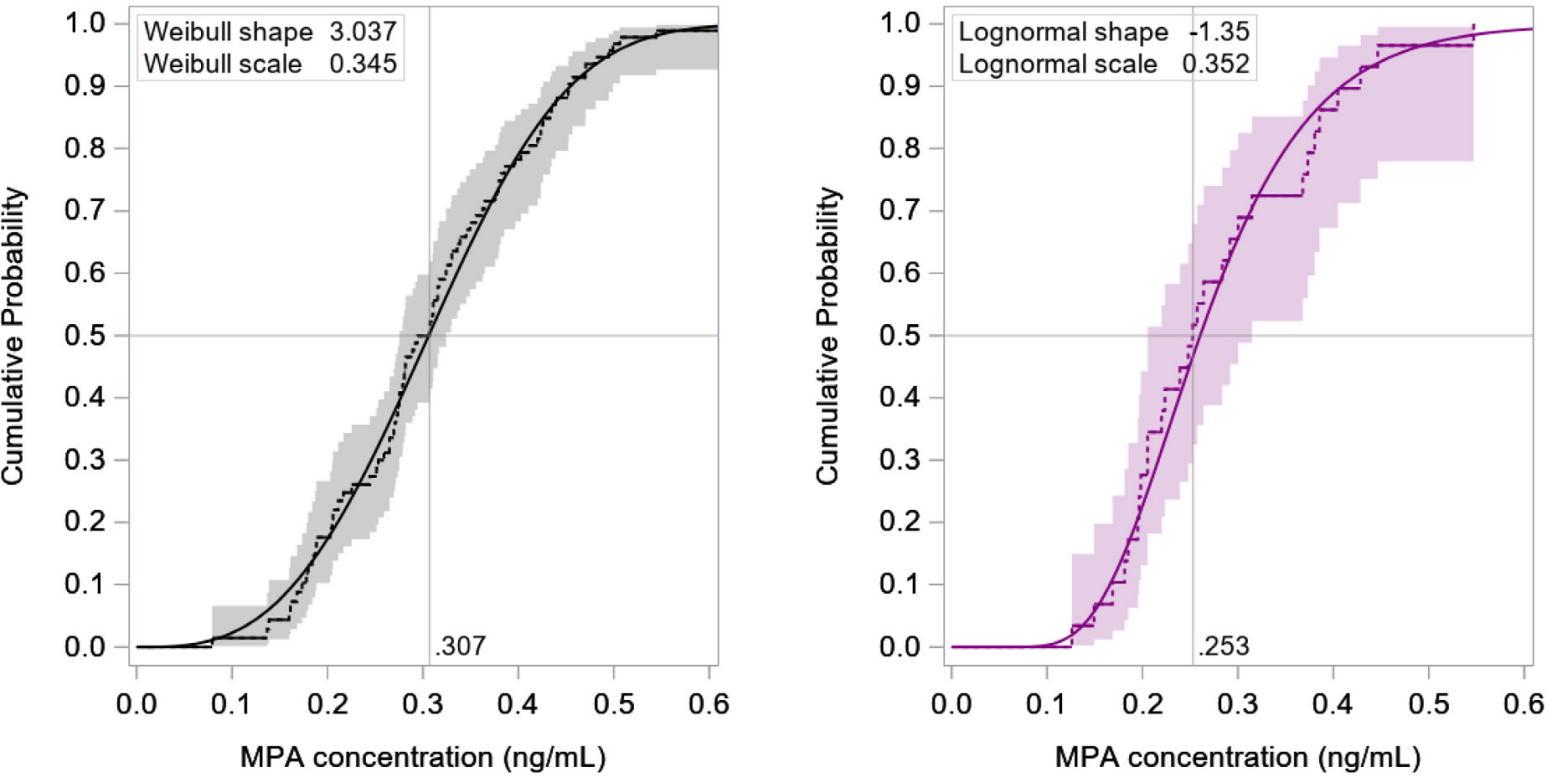
Cumulative distribution of MPA concentrations 4 months after a 104 mg subcutaneous injection (n = 95; left) and seven months after a 150 mg subcutaneous injection (n = 29; right), based on data from two trials conducted between 2015 and 2018 [10,11]. Dashed lines and 95% confidence bands are based on non-parametric maximum likelihood estimation. Solid curves are parametric model fits. Abbreviation: MPA, medroxyprogesterone acetate.

We included all 95 evaluable participants in all dose groups (normalized to 104 mg) when estimating the cumulative distribution of MPA levels 4 months after a 104 mg subcutaneous injection (*X_m4_*). The nonparametric estimate of the median concentration at month 4 was 0.31 ng/mL (95% CI: 0.28–0.32) and 90% of participants exceeded 0.17 ng/mL (95% CI: 0.14–0.19; Fig. 4). A sensitivity analysis restricted to just the 39 participants in the 104 mg and 105 mg groups led to similar results (median: 0.30 ng/mL; 10th percentile: 0.18 ng/mL).

We only included the 29 evaluable participants in the 150 mg and 300 mg groups (normalized to 150 mg) when estimating the cumulative distribution of MPA levels 7 months after a 150 mg subcutaneous injection (*X_m7_*), owing to the lack of dose-proportionality in the lower dose groups. The estimated median concentration was 0.25 ng/mL (95% CI: 0.20–0.30) and 90% of participants exceeded 0.17 ng/mL (95% CI: 0.00–0.20; Fig. 4). Restricting the analysis to just the 21 evaluable participants in the 150 mg group led to similar results (median: 0.25 ng/mL; 10th percentile: 0.18 ng/mL). Neither age nor race was significantly associated with the distribution of MPA at month 4 or 7. Body mass index greater than 30.0 was associated with lower MPA concentrations at month 4, but only in a sensitivity analysis restricted to the 39 participants who received a 104 mg or 105 mg dose (of whom only 6 were in the high BMI category; Supplemental Table S3).

We estimated the probability of ovulation within 4 months of a 104 mg subcutaneous injection to be 1.5% when randomly sampling 50,000 replicates from the empirical distributions of *Y_ov_* (Fig. 3) and X_m4_ (Fig. 4) to solve Eq. (1). Assuming these were each distributed as Weibull random variables led to a similar result (1.3%; 95% bootstrap CI: 0.5–2.3). The estimated probability of ovulation within 7 months of a 150 mg subcutaneous injection was 2.1% when randomly sampling from the empirical distributions of *Y_ov_* and X_m7_, and 0.4% (95% CI: 0.1–1.4) when assuming these variables were distributed Weibull and log-normal, respectively (the discrepancy between the latter non-parametric and parametric solutions may be attributed to a conservative assumption made when sampling from the empirical distribution of *X_m7_*; see supplemental Appendix).

## 4 Discussion

Prescribing information for Depo-subQ emphasizes the need to adhere to a 3-month dosing schedule [4]. Although ovulations can still occur, such events are rare, and no pregnancies were detected in phase 3 trials of the regimen. In our pharmacokinetics and pharmacodynamics studies, 0 of 30 participants who received a single 104 mg or 105 mg subcutaneous injection of MPA experienced an elevated serum progesterone concentration ≥4.7 ng/mL before month 5.5, and 0 of 21 who received a 150 mg subcutaneous injection ovulated by this criterion before month 7.5 (qualitatively similar findings were obtained using a progesterone threshold of 3.0 ng/mL; results not shown). Our modeling results that leveraged data from all (45–300 mg) dose groups were consistent with the empirical evidence: less than a 2.2% chance of ovulation within four months of a 104 mg subcutaneous injection of MPA or within 7 months of a 150 mg subcutaneous injection of MPA. We also estimated that 90% of women do not ovulate until their serum MPA concentration falls below 0.10 ng/mL, and 50% do not ovulate until their MPA level falls below 0.07 ng/mL. These results emphasize that the commonly cited MPA threshold of 0.2 ng/mL is too high for the majority of women, and that the 0.1 ng/mL level noted by others is a more reasonable guidance [6,15].

Our study results are consistent with some but not all historical trials of Depo-subQ. We observed geometric mean C_max_ values of 0.80 to 0.82 ng/mL in the 104 mg and 105 mg dose groups that were lower than the mean (median) of 1.56 (1.49) ng/mL reported in the Depo-subQ label study [5,6]. We also observed a more extended drug release profile and a longer delay in return to ovulation than in the label study (Supplemental Fig. S3). Although population differences may partially explain this, site of injection could also play a role. The injection site is not noted in references for the label study, while we exclusively gave subcutaneous injections in the abdomen. The latter was associated with a lower C_max_ than injections in the thigh in another trial [7]. On the other hand, our results are consistent with a comparative study of 64 women who received subcutaneous injections of Depo-subQ (104 mg MPA in glass syringe) or Sayana Press (104 mg MPA in the Uniject device) [8]. That trial reported a GM C_max_ of 0.79 ng/mL for Depo-subQ which is essentially identical to our result. And although there was one ovulation before month 3 in each device group (excluding women with dose administration errors), none occurred between 3 and 5 months of injection. Some differences between contemporary and historical studies could arise due to overfill of the Depo-subQ glass syringe, which may not have been used in trials that predate approval of the product [11]. We conducted sensitivity analyses that accounted for up to 10% overfill, but the results did not meaningfully change. However, the predicted MPA concentration 6 months after a 150 mg dose was equivalent to the level 3 months after a 104 mg dose when adjusting for overfill (GM ratio: 0.94; 90% CI: 0.80–1.11), lending further support to a 6-month 150 mg regimen.

There are several limitations to our analysis, most importantly the absence of participants with class II+ obesity (BMI ≥35). We did not observe consistent trends between risk of ovulation and BMI in the range of 18 to 34, which agrees with earlier reports [6]. However, this does not mean our results necessarily apply to women with more extreme BMI. To the best of our knowledge the relationship between pharmacokinetics and pharmacodynamics of MPA in class II+ obesity has only been formally assessed in one study, making this a valuable area of future research [16]. A second limitation is the racial diversity of our study participants, who primarily identified as Caucasian or biracial of African and European descent. Although the pharmacokinetics and pharmacodynamics of Depo-subQ is reported to be similar for Caucasian, African American, and Asian women, we cannot rule out important differences among other racial groups [4]. Pharmacogenetic research to identify factors that impact the pharmacokinetics or pharmacodynamics of MPA and their potential associations with geographic region, race or ethnicity is another important area of future research. A third limitation is that the MPA level associated with elevation of progesterone ≥4.7 ng/mL is expected to be somewhat lower than the level required to suppress ovulation due to the delay between these events. Our observation that women with faster rates of MPA decline tended to have lower drug levels when progesterone became elevated is consistent with this delay. In a sensitivity analysis, however, the median MPA level ten days prior to when progesterone became elevated was still only 0.08 ng/mL (90th percentile: 0.12 ng/mL). Finally, we implicitly extrapolated beyond the range of the data when modeling the probability of rare outcomes (e.g., ovulation within 4 months of a 104 mg or 105-mg injection, when no such events occurred). Fully parametric population PK/PD models could increase precision in this setting but may not perform well when assumptions underlying them are miss-specified [17]. We tried to minimize model assumptions by using nonparametric methods but cannot rule out biases.

It is distinctly possible that any real-world Depo-subQ contraceptive failures are disproportionately attributable to issues largely unrelated to dose, such as mistimed treatment initiation or inadvertent injection near a vascular site. The use of an unnecessarily high exposure to limit the residual chance of treatment failure would be a disservice to the vast majority of women if a lower exposure can reduce side effects, costs, or otherwise make the product more acceptable. Our secondary analysis reinforces conclusions in one of the constituent study manuscripts that there would be little risk of pregnancy if the Depo-subQ reinjection interval were extended from 3 to 4 months [11]. This has since been confirmed in an efficacy trial that observed a pregnancy rate of 0.00 per 100 women-years (95% CI: 0.00–0.59) among 750 women injecting Sayana Press every four months [18]. A 150mg subcutaneous injection of Depo-Provera every 6 months (with up to a 1-month grace period) also appears highly effective, could be more acceptable due to less frequent injections, and would reduce long-term MPA exposure by 28% compared to the existing Depo-subQ regimen.

## Appendix Supplementary materials

Image, application 1

Image, application 2
