# Supplementary material for: Ovulation suppression following subcutaneous administration of depot medroxyprogesterone acetate
Source: Contracept X. 2022 Feb 23;4:100073. doi: 10.1016/j.conx.2022.100073 (PMC8907671; doi:10.1016/j.conx.2022.100073)
Supplement: Supplementary file 1 [file mmc1.docx]

**Supplemental Appendix**

This Appendix provides additional details regarding study data, estimation methods, and results of supplemental analyses.

***Data handling***

The primary analysis of Study 702179 [10] censored data from one subject in the 150mg Depo-Provera group on treatment day 70 due to the initiation of a concomitant medication (meloxicam) which could impact ovulation function. We did not censor this data from our secondary analysis since the medication was only used for 5 days and the subject was not otherwise at risk of ovulation that early in follow-up.

The primary analysis of Study 834119 [11] censored data from one subject on treatment day 5 due to the use meloxicam for the duration of their follow-up. We entirely excluded this subject from our analysis of MPA concentrations when ovulation returned.

Two subjects in Study 834119 had an elevated progesterone within 14 days of treatment initiation (one each in the 45mg and 75mg Depo-Provera groups). These subjects were excluded from the estimation of MPA concentrations when ovulation returned, since either their MPA exposure was not sufficient to initially suppress ovulation, or they received a mistimed injection in relation to start of menses.

The primary pharmacokinetics analyses of Study 702179 and Study 834119 each excluded three subjects with baseline MPA levels that exceeded 5% of their individual C_max_ (3, 2, and 1 in the 75mg, 150mg , and 300mg Depo-Provera groups, respectively). We excluded these subjects from our descriptive pharmacokinetic analyses but not when estimating the distribution of MPA concentrations when ovulation returned.

We used retest MPA values for four outliers in Study 834119 (one in each dose group), leading to GM C_max_ values that were approximately 0.05 ng/mL lower than those reported in the primary manuscript.

***Computing the distribution of MPA concentrations when ovulation returned***

We estimated the pharmacokinetic profile of each subject, from day of treatment initiation through the last day of MPA sampling, using locally re-weighted non-parametric regression with the SAS/STAT LOESS procedure. We used cubit interpolation when blending local polynomials, restricting estimation of the smoothing parameter to between 0.1 and 0.6. We subsequently obtained the predicted MPA concentration on the last day that progesterone was not elevated (M_ne_) and the first day that an elevated progesterone was detected (M_e_) for each subject. If no elevated progesterone was observed, then M_e_ was missing (left-censored). Three subjects who stopped contributing MPA data between 8 and 15 days prior to their last non-elevated progesterone had M_ne_ imputed based on their apparent terminal half-life.

The cumulative distribution of MPA concentrations when ovulation returned was estimated using the SAS ICLIFETEST procedure to implement Turnbull’s non-parametric maximum likelihood algorithm, applied to the intervals [M_e_, M_ne_]. This is analogous to performing a survival analysis, but with the time scale replaced by MPA concentration. 95% CIs were obtained using the log-log transformation of the survival function. The Weibull distribution was selected as the best parametric alternative based on having the minimum Bayesian Information Criteria among the following distributions: Weibull, log-normal, log-logistic, gamma, and Weibull with Gamma frailty.

***Computing the probability of ovulation within 4 or 7 months of a 104mg or 150mg injection***

We approximated the probability of ovulation within *T* months of a dose *D* based on the following:

$Pr(\text{ovulate on or before month }T)=\int_{0}^{\infty} F_{X}(y)dF_{Y}(y).$ (A.1)

Here, *F_X_*(*·*) is the cumulative distribution of MPA concentrations at month *T* (denoted *X_T_*) and *F_Y_*(*·*) is the distribution of the MPA concentrations when ovulation returned (*Y_ov_*). Parametric solutions to equation A.1 were obtained using the SAS/IML QUAD function to perform the necessary numerical integration, with 95% CIs computed based on 10,000 bootstrap samples (re-estimating *F_X_*(*·*) and *F_Y_*(*·*) with each sample).

Non-parametric solutions were obtained in a two-step process. First, we randomly sampled 50,000 replicates of *X_T_* from the Turnbull estimate of *F_X_*(*·*), assuming *X_T_* was uniformly distributed within each step of the empirical distribution function. Then we averaged the estimated probabilities that *Y_ov_* exceeded *x_T_* based on the Turnbull estimate of *F_Y_*(∙).

Discreteness in the data may have led us to perceptibly over-estimate the probability of ovulation within 7 months of a 150mg injection (2.1%). There were only 29 observations with which to estimate the distribution *X_m7_*, resulting in 3.4% of the random sample of 50,000 values falling uniformly between 0.0 and 0.126 ng/mL (the first ‘jump’ in the empirical distribution of *X_m7_*). This uniformity assumption meant that MPA values close to 0.0 (virtually guaranteed to result in ovulation) were just as likely to be sampled as values close to 0.126 (little risk of ovulation). Had we assumed that higher drug levels are more likely than lower drug levels in the range 0.0-0.126 ng/mL, then our estimated probability of ovulation would have been less than 2.1%.

***Key assumptions underlying Equation A.1:***

- *X_T_* is independent of *Y_ov_*:

Violations of this assumption would be apparent if the distribution of *Y_ov_* depended on administered dose of MPA (which directly impacts *X_T_*), but we found no evidence of such an effect.

- *Y_ov_* does not vary with time:

There is limited data to assess the validity of this assumption, since so few subjects were followed after their first ovulation (and none who received doses over 75mg). As noted above, there was no apparent relationship between extent (and duration) of exposure and the distribution of *Y_ov_*, but we cannot rule out the possibility that systematic effects of time or exposure exist.

- MPA concentrations uniformly decrease during periods when a risk of ovulation exists (i.e., there is no time *Z* < *T* such that Pr{*X_Z_* < *Y_ov_* < *X_T_*} > 0 ):

MPA concentrations may not uniformly decrease with time, and so it is possible for an individual to have drug levels below their *y_ov_* value at some point before month *T*, only to exceed *y_ov_* at month *T*. Thus, Equation A.1 under-estimates the risk of ovulation on or before month *T* by Pr(*X_min_ < Y_ov_ < X_T_*), where *X_min_* is the minimum concentration on or before month *T*. As evident from supplemental Table S2, however, the distribution of *X_min_* was not meaningfully different from the distribution of *X_T_* for the MPA formulations, doses (104mg and 150mg) and time points (month 4 and 7) of interest here (after excluding the first 7 days of treatment, when MPA levels are increasing from zero). Hence, bias should be small. This was demonstrated in sensitivity analyses which led to essentially identical (+/- 0.1%) estimated probabilities of ovulation within 4 months of a 104mg dose or within 7 months of a 150mg dose when replacing *X_T_* with *X_min_* in Equation A.1.


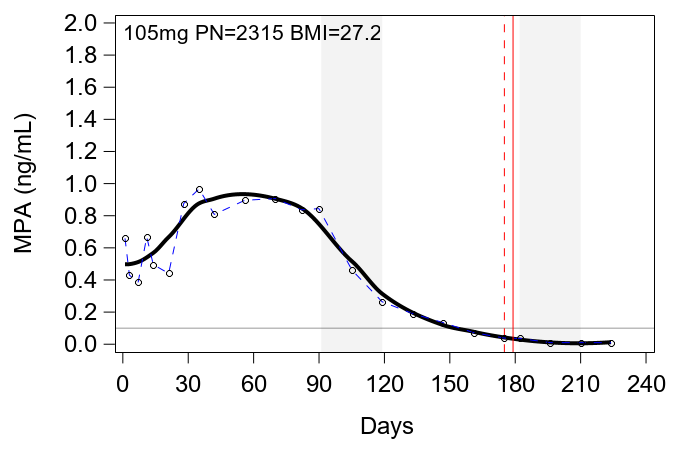

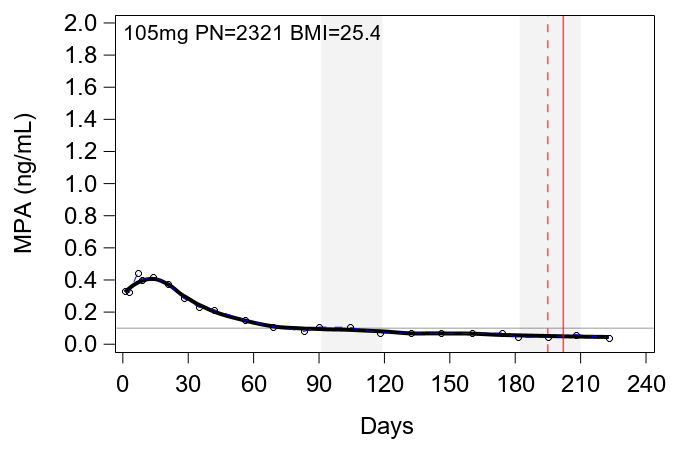

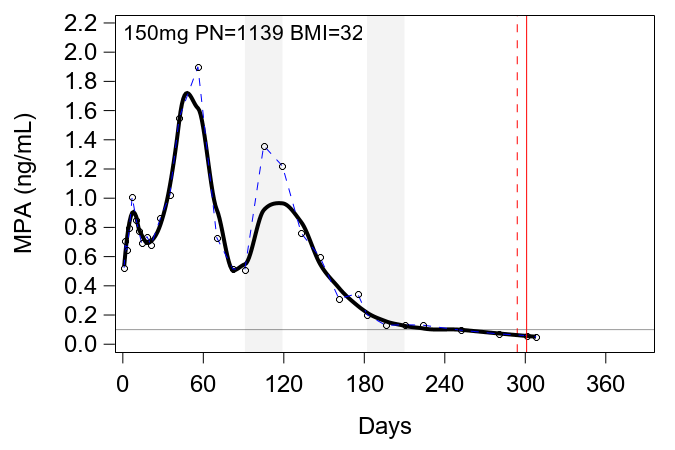

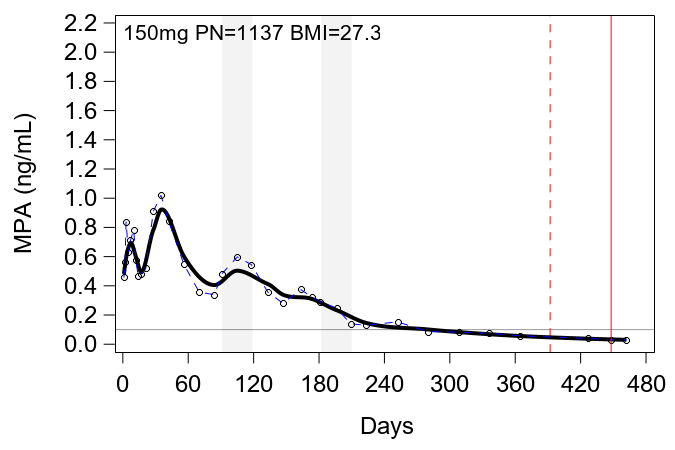


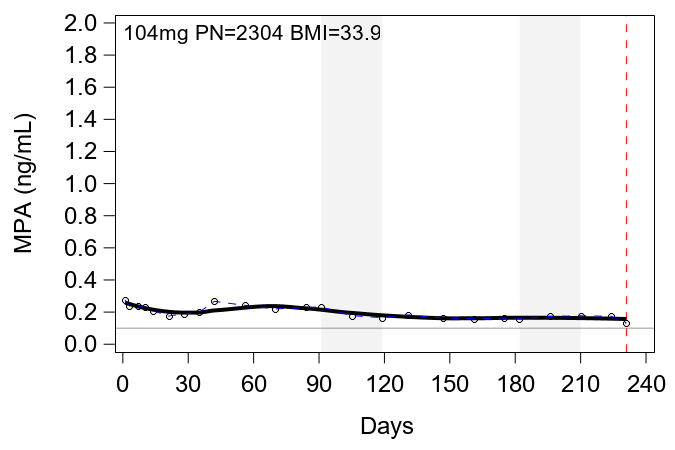

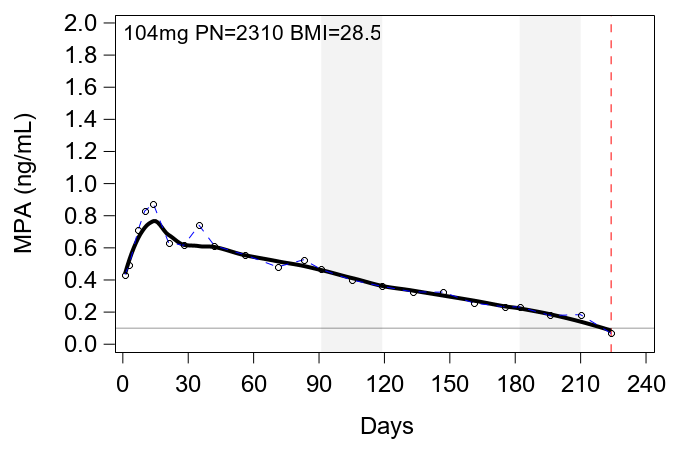

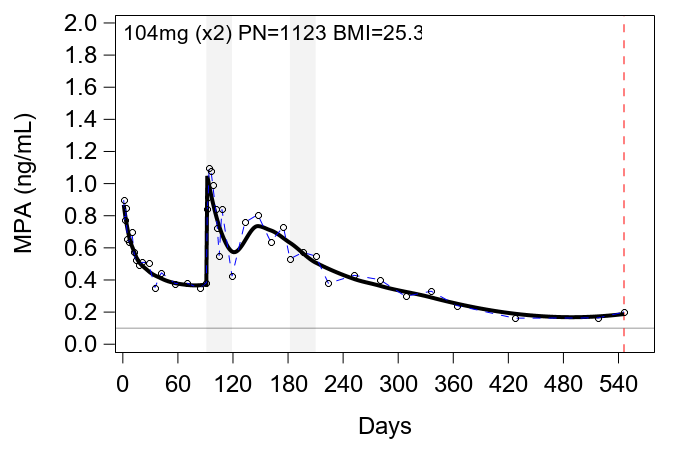

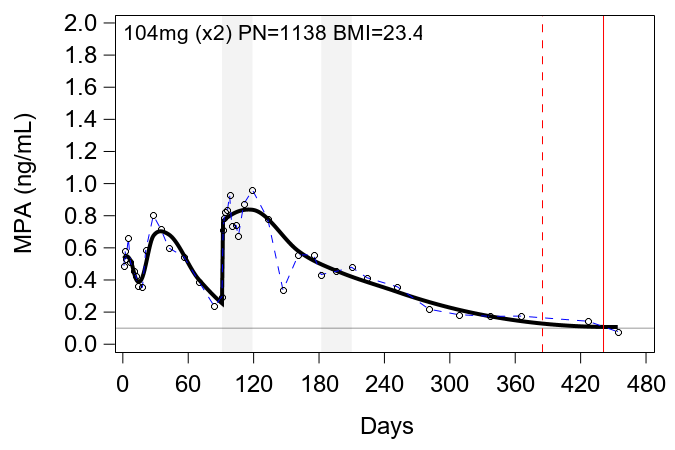


**Supplemental Figure S1**. Example PK profiles for a single 105 or 150mg subcutaneous injection of Depo-Provera (top rows), and one (x1) or two (x2) 104mg injections of Depo-subQ (bottom rows). Dashed and solid vertical lines are day of last non-elevated and first elevated progesterone ≥ 4.7 ng/mL.


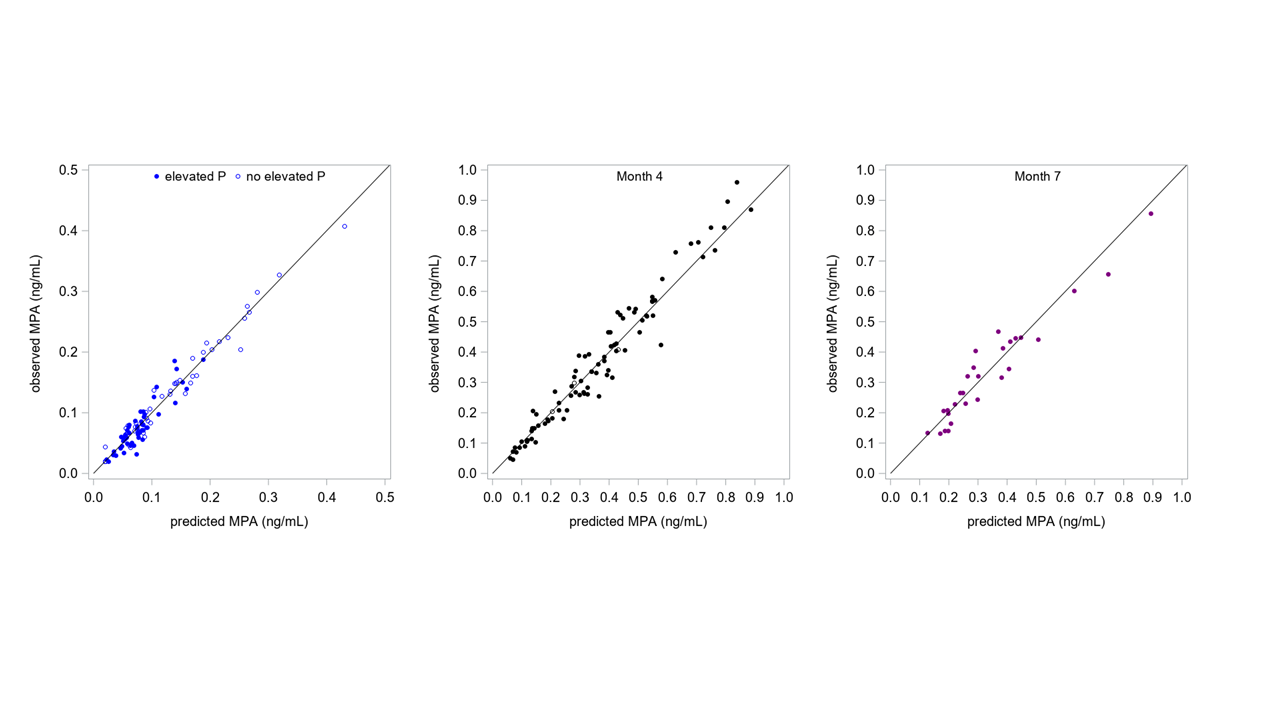


**Supplemental Figure S2**. Observed versus LOESS-predicted MPA concentrations when ovulation returns (left), four months after a 104mg subcutaneous injection (dose-normalized 45-300mg data; middle), and seven months after a 150mg subcutaneous injection (dose-normalized 150-300mg data; right).


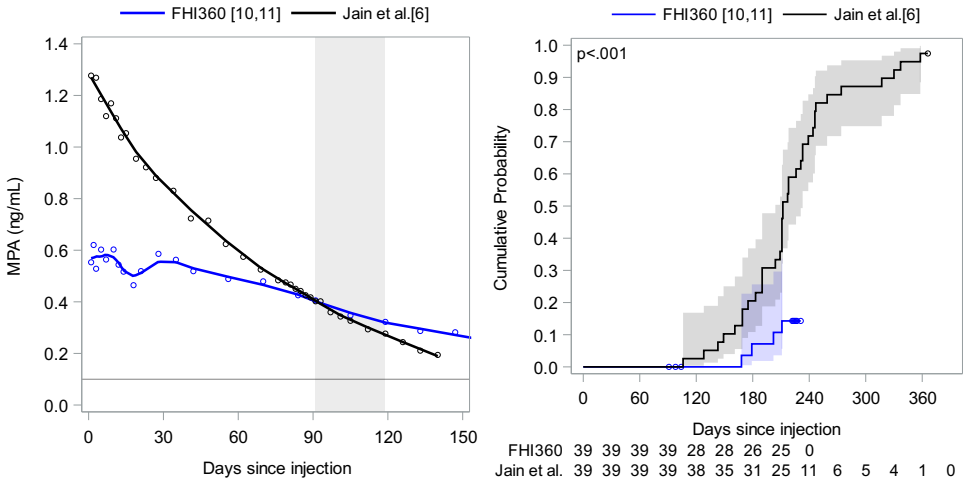


**Supplemental Figure S3. Left:** arithmetic mean MPA concentrations in the 104-105mg dose groups in our studies and digitalized from the Depo-subQ label [6,10,11]. **Right:** corresponding cumulative probabilities of ovulation (progesterone ≥ 4.7 ng/mL), with numbers at risk below the x-axis.
