# Supplementary material for: Ovulation suppression following subcutaneous administration of depot medroxyprogesterone acetate
Source: Contracept X. 2022 Feb 23;4:100073. doi: 10.1016/j.conx.2022.100073 (PMC8907671; doi:10.1016/j.conx.2022.100073)
Supplement: Supplementary file 2 [file mmc2.docx]

**Supplemental Table S1**. Geometric mean predicted MPA concentrations (ng/mL) and 95% CIs^†^ following subcutaneous administration of 45-300mg Depo-Provera or 104mg Depo-subQ in two trials conducted between 2015 and 2018 [10,11]

|  | **45mg**  **(n=15)** | **75mg**  **(n=12)** | **104mg**  **(n=24^‡^)** | **105mg**  **(n=15)** | **Pooled 104/105 (n=39^‡^)** | **150mg**  **(n=21)** | **300mg**  **(n=8)** | **p-value**^¥^ |
| --- | --- | --- | --- | --- | --- | --- | --- | --- |
| C_max_ | 0.35 (0.27, 0.46) | 0.55 (0.38, 0.80) | 0.80 (0.68, 0.93) | 0.82 (0.72, 0.93) | 0.80 (0.72, 0.89) | 1.12 (0.98, 1.27) | 1.74 (1.37, 2.20) | <0.001 |
| C_mo3_ | 0.14 (0.11, 0.17) | 0.26 (0.18, 0.37) | 0.37 (0.32, 0.43) | 0.34 (0.26, 0.44) | 0.36 (0.32, 0.41) | 0.54 (0.47, 0.62) | 1.04 (0.78, 1.37) | <0.001 |
| C_mo4_ | 0.11 (0.09, 0.14) | 0.21 (0.15, 0.30) | 0.32 (0.27, 0.37) | 0.28 (0.22, 0.37) | 0.30 (0.26, 0.35) | 0.48 (0.42, 0.56) | 0.87 (0.67, 1.13) | <0.001 |
| C_mo5_ | 0.09 (0.07, 0.11) | 0.16 (0.12, 0.23) | 0.27 (0.23, 0.32) | 0.21 (0.16, 0.29) | 0.24 (0.20, 0.28) | 0.40 (0.35, 0.45) | 0.76 (0.61, 0.95) | <0.001 |
| C_mo6_ | 0.06 (0.05, 0.08) | 0.12 (0.08, 0.18) | 0.23 (0.20, 0.26) | 0.15 (0.10, 0.22) | 0.18 (0.15, 0.23) | 0.32 (0.28, 0.36) | 0.68 (0.53, 0.88) | <0.001 |
| C_mo7_ | 0.04 (0.03, 0.06) | 0.09 (0.06, 0.13) | 0.18 (0.15, 0.21) | 0.10 (0.06, 0.18) | 0.14 (0.10, 0.18) | 0.25 (0.22, 0.29) | 0.58 (0.40, 0.83) | <0.001 |
|  | **Normalized to 104mg** | | | | | | |  |
| C_max_ | 0.82 (0.63, 1.06) | 0.76 (0.52, 1.11) | 0.80 (0.68, 0.93) | 0.81 (0.71, 0.92) | 0.80 (0.72, 0.89) | 0.77 (0.68, 0.88) | 0.60 (0.48, 0.76) | 0.067 |
| C_mo3_ | 0.32 (0.25, 0.40) | 0.36 (0.25, 0.52) | 0.37 (0.32, 0.43) | 0.34 (0.26, 0.44) | 0.36 (0.32, 0.41) | 0.37 (0.33, 0.43) | 0.36 (0.27, 0.47) | 0.508 |
| C_mo4_ | 0.26 (0.21, 0.32) | 0.30 (0.21, 0.42) | 0.32 (0.27, 0.37) | 0.28 (0.22, 0.36) | 0.30 (0.26, 0.34) | 0.34 (0.29, 0.38) | 0.30 (0.23, 0.39) | 0.281 |
| C_mo5_ | 0.20 (0.16, 0.25) | 0.23 (0.16, 0.32) | 0.27 (0.23, 0.32) | 0.21 (0.16, 0.28) | 0.24 (0.20, 0.28) | 0.28 (0.24, 0.31) | 0.26 (0.21, 0.33) | 0.059 |
| C_mo6_ | 0.14 (0.11, 0.19) | 0.17 (0.11, 0.25) | 0.23 (0.20, 0.26) | 0.14 (0.10, 0.22) | 0.18 (0.14, 0.23) | 0.22 (0.19, 0.25) | 0.24 (0.18, 0.30) | 0.008 |
| C_mo7_ | 0.10 (0.07, 0.14) | 0.13 (0.09, 0.18) | 0.18 (0.15, 0.21) | 0.10 (0.06, 0.17) | 0.13 (0.10, 0.18) | 0.17 (0.15, 0.20) | 0.20 (0.14, 0.29) | 0.004 |

^†^ Maximum (C_max_) based on observed values. Concentrations at specified months (C_mo_) based on LOESS-predicted levels at the specified month.

^‡^ Data from 9 subjects in the 104mg group who received a second injection at month 3 are excluded from calculations at month 4 and onwards.

^¥^ Based on test of no trend in mean logged response with respect to dose of MPA.

**Supplemental Table S2**. Geometric mean predicted minimum MPA concentrations (ng/mL) on or before a given month^†^ (and 95% CIs) following subcutaneous administration of 45-300mg Depo-Provera or 104mg Depo-subQ in two trials conducted between 2015 and 2018 [10,11]

|  | **45mg**  **(n=15)** | **75mg**  **(n=12)** | **104mg**  **(n=24^‡^)** | **105mg**  **(n=15)** | **Pooled 104/105 (n=39^‡^)** | **150mg**  **(n=21)** | **300mg**  **(n=8)** | **p-value**^¥^ |
| --- | --- | --- | --- | --- | --- | --- | --- | --- |
| C_mo3_ | 0.13 (0.10, 0.16) | 0.22 (0.16, 0.31) | 0.34 (0.30, 0.39) | 0.31 (0.24, 0.40) | 0.33 (0.29, 0.37) | 0.46 (0.41, 0.53) | 0.81 (0.60, 1.09) | <0.001 |
| C_mo4_ | 0.11 (0.09, 0.13) | 0.20 (0.15, 0.27) | 0.31 (0.27, 0.36) | 0.27 (0.21, 0.35) | 0.29 (0.25, 0.34) | 0.42 (0.37, 0.48) | 0.74 (0.57, 0.96) | <0.001 |
| C_mo5_ | 0.08 (0.07, 0.10) | 0.16 (0.12, 0.23) | 0.27 (0.23, 0.31) | 0.20 (0.16, 0.27) | 0.23 (0.20, 0.27) | 0.37 (0.33, 0.42) | 0.67 (0.55, 0.82) | <0.001 |
| C_mo6_ | 0.06 (0.05, 0.08) | 0.12 (0.08, 0.18) | 0.23 (0.20, 0.26) | 0.14 (0.10, 0.21) | 0.18 (0.14, 0.22) | 0.31 (0.27, 0.35) | 0.60 (0.49, 0.73) | <0.001 |
| C_mo7_ | 0.04 (0.03, 0.06) | 0.09 (0.06, 0.13) | 0.18 (0.15, 0.21) | 0.10 (0.06, 0.17) | 0.13 (0.10, 0.18) | 0.25 (0.21, 0.28) | 0.52 (0.39, 0.69) | <0.001 |
|  | **Normalized to 104mg** | | | | | | |  |
| C_mo3_ | 0.30 (0.23, 0.37) | 0.31 (0.23, 0.42) | 0.34 (0.30, 0.39) | 0.31 (0.24, 0.40) | 0.33 (0.29, 0.37) | 0.32 (0.28, 0.37) | 0.28 (0.21, 0.38) | 0.641 |
| C_mo4_ | 0.25 (0.20, 0.30) | 0.28 (0.20, 0.38) | 0.31 (0.27, 0.36) | 0.27 (0.21, 0.35) | 0.29 (0.25, 0.33) | 0.29 (0.26, 0.33) | 0.26 (0.20, 0.33) | 0.890 |
| C_mo5_ | 0.19 (0.16, 0.24) | 0.23 (0.16, 0.32) | 0.27 (0.23, 0.31) | 0.20 (0.16, 0.27) | 0.23 (0.20, 0.27) | 0.26 (0.23, 0.29) | 0.23 (0.19, 0.28) | 0.206 |
| C_mo6_ | 0.14 (0.11, 0.19) | 0.17 (0.11, 0.25) | 0.23 (0.20, 0.26) | 0.14 (0.09, 0.21) | 0.18 (0.14, 0.22) | 0.21 (0.19, 0.24) | 0.21 (0.17, 0.25) | 0.038 |
| C_mo7_ | 0.10 (0.07, 0.14) | 0.12 (0.08, 0.18) | 0.18 (0.15, 0.21) | 0.10 (0.06, 0.17) | 0.13 (0.10, 0.18) | 0.17 (0.15, 0.20) | 0.18 (0.14, 0.24) | 0.012 |

^†^ Excluding the first 7 days of treatment, during which time MPA concentrations may still have been increasing from zero.

^‡^ Data from 9 subjects in the 104mg group who received a second injection at month 3 are excluded from calculations at month 4 and onwards.

^¥^ Based on test of no trend in mean logged response with respect to dose of MPA.

**Supplemental Table S3.** Median predicted MPA concentrations (ng/mL) by participant-level factors, based on modelling of data from two trials of subcutaneously administered Depo-Provera and Depo-subQ conducted between 2015 and 2018 (Month 4 and Month 7 results based on Weibull and log-normal distribution for dose-normalized data, respectively)^†^ [10,11]

|  |  | Month 4 (104 mg dose; n=95) | |  | Month 7 (150 mg dose; n=29) | |
| --- | --- | --- | --- | --- | --- | --- |
|  | n | Median (95% CI) | p-value | n | Median (95% CI) | p-value |
| **BMI (kg/m2)** |  |  | 0.37^‡^ |  |  | 0.32 |
| < 25 | 30 | 0.312 (0.277, 0.353) ^‡^ |  | 9 | 0.247 (0.198, 0.308) |  |
| 25-30 | 42 | 0.292 (0.262, 0.325) ^‡^ |  | 11 | 0.243 (0.199, 0.300) |  |
| > 30 | 23 | 0.327 (0.283, 0.378) ^‡^ |  | 9 | 0.300 (0.240, 0.374) |  |
| **Age (years)** |  |  | 0.15 |  |  | 0.06 |
| ≤ 35 | 58 | 0.296 (0.270, 0.324) |  | 20 | 0.281 (0.234, 0.335) |  |
| > 35 | 37 | 0.327 (0.290, 0.367) |  | 9 | 0.220 (0.183, 0.265) |  |
| **Race** |  |  | 0.47 |  |  | 0.57 |
| Black/biracial | 62 | 0.300 (0.273, 0.330) |  | 27 | 0.263 (0.230, 0.300) |  |
| White | 33 | 0.316 (0.280, 0.360) |  | 2 | 0.228 (0.140, 0.370) |  |

^†^ Month 7 results were restricted to the 150 and 300mg groups due to lack of proportionality at lower doses

^‡^ When restricting the analysis to n=39 subjects in the 104mg and 105mg dose groups, median MPA levels were 0.322 (n=13), 0.309 (n=20), and 0.218 ng/mL (n=6) in the BMI<25, 25<BMI<30, and BMI>30 strata (p=0.01).
